# Experiences of shared decision-making in community rehabilitation: a focused ethnography

**DOI:** 10.1186/s12913-020-05223-4

**Published:** 2020-04-19

**Authors:** Kiran Pohar Manhas, Karin Olson, Katie Churchill, Sunita Vohra, Tracy Wasylak

**Affiliations:** 1 c/o Strategic Clinical Networks™, Alberta Health Services, Southport Tower, 10301 Southport Lane SW, Calgary, Alberta T2W 1S7 Canada; 2 Integrative Health Institute, University of Alberta, Edmonton, Alberta Canada; 3 Faculty of Nursing, University of Alberta, Edmonton, Alberta Canada; 4 Department of Clinical Neurosciences, Cumming School of Medicine, University of Calgary, Calgary, Alberta Canada; 5 Department of Occupational Therapy, University of Alberta, Edmonton, Alberta Canada; 6 Departments of Pediatrics and Psychiatry, Faculty of Medicine & Dentistry, University of Alberta, Edmonton, Alberta Canada; 7 Faculty of Nursing, University of Calgary, Calgary, Alberta Canada

**Keywords:** Shared decision-making, Community, Rehabilitation, Patient perspectives, Provider perspectives, Expectations, Buy-in

## Abstract

**Background:**

Shared decision-making (SDM) can advance patient satisfaction, understanding, goal fulfilment, and patient-reported outcomes. We lack clarity on whether this physician-focused literature applies to community rehabilitation, and on the integration of SDM policies in healthcare settings. We aimed to understand patient and provider perceptions of shared decision-making (SDM) in community rehabilitation, particularly the barriers and facilitators to SDM.

**Methods:**

We used a focused ethnography involving 14 community rehabilitation sites across Alberta, including rural, regional-urban and metropolitan-urban sites. We conducted semi-structured interviews that asked participants about their positive and negative communication experiences (*n* = 23 patients; *n* = 26 providers).

**Results:**

We found SDM experiences fluctuated between extremes: Getting Patient Buy-In and Aligning Expectations. The former is provider-driven, prescriptive and less flexible; the latter is collaborative, inquisitive and empowering. In Aligning Expectations, patients and providers express humility and openness, communicate in the language of ask and listen, and view education as empowering. Patients and providers described barriers and facilitators to SDM in community rehabilitation. Facilitators included geography influencing context and connections; consistent, patient-specific messaging; patient lifestyle, capacity and perceived outlook; provider confidence, experience and perceived independence; provider training; and perceptions of more time (and control over time) for appointments. SDM barriers included lack of privacy; waitlists and financial barriers to access; provider approach; how choices are framed; and, patient’s perceived assertiveness, lack of capacity, and level of deference.

**Conclusions:**

We have found both excellent experiences and areas for improvement for applying SDM in community rehabilitation. We proffer recommendations to advance high-quality SDM in community rehabilitation based on promoting facilitators and overcoming barriers. This research will support the spread, scale and evaluation of a new Model of Care in rehabilitation by the provincial health system, which aimed to promote patient-centred care.

## Background

Globally, nationally and provincially, health-systems and research-funders aim to promote patient-centred care [1–3]. Patient-centered care is defined as respectful, responsive care that incorporates patient needs and values [4]. Shared decision-making (SDM) is an interpersonal process where provider(s) and patient collaborate to make decisions using best available evidence as well as patient preferences and lived experience [2–10]. Both patient-centered care and SDM are integral components of enhanced patient experience.

Research suggests that SDM advances patient knowledge and satisfaction [11–13], promotes the attainment of treatment goals [14], reduces inappropriate service utilization [15], and improves patient-reported outcomes [11, 16]. SDM is not routinely used or taught in healthcare [17–19]. Systematic reviews (professionals (*n* = 38 [18], *n* = 20 [20] studies), and patients (*n* = 44 [21] studies)) suggest that SDM barriers include time-related barriers, organizational facets (e.g. lack of recognition and reimbursement), traits of the interaction (e.g. power imbalance), and patient characteristics (e.g. lack agreement) [18, 20, 21]. SDM facilitators include attitudes, patient preferences, and level of innovation [18].

The extant SDM literature emphasizes patient-physician interactions. Little research exists on the experience and impacts of SDM amongst other professionals, teams and organizations [22]; and on SDM in rehabilitation involving primarily allied-health providers [23]. A narrative synthesis (*n* = 15 studies) revealed that in-patient rehabilitation goal-setting did not permit patient input, was overly-controlled by staff, was challenging for time and patient-load reasons, and involved parties lacking SDM knowledge [23]. Five further studies evaluated a “train-the-trainer” program to promote SDM in inpatient rehabilitation using focus groups, surveys and a cluster-randomized controlled study, but did not fully elaborate the SDM experience in rehabilitation [24–28]. Other research theorizes on SDM in rehabilitation, positing on technology, ethics and collaboration [29–33]. Study transferability to community contexts is unclear [23]. Inpatient and outpatient needs and resources vary, impacting communication [34, 35]. More research is required to better understand the barriers and facilitators to SDM in a rehabilitation context, particularly because rehabilitation supports both acute and chronic care. This better understanding is timely, relevant and important.

In this study, we aimed to address this gap in understanding the experience of SDM in community rehabilitation. We sought to understand what patients and providers from diverse community-rehabilitation sites across one Canadian province perceived as the SDM experience, its barriers and facilitators. We conducted a feasibility study with two community-rehabilitation sites, wherein strategies were developed to address site and participant burden; to promote recruitment success; and, to confirm the appropriateness of data collection techniques.

## Methods

Using focused ethnography, we studied patients and professionals composing diverse outpatient rehabilitation sites across a Canadian province (population 4 million people), whether hospital- or community- based. We used multiple data collection techniques including prospective surveys and informant interviews; we discuss the qualitative findings here only. The research team initially reflexively noted their own assumptions, which included beliefs that SDM results in better outcomes, that micro-, meso- and macro- level factors influenced SDM; and that this research is “real-world.” Recognition of these beliefs led to emphasis on audit trail and study limitation discussions; asking providers questions about team (meso) and organizational (macro) factors as well as the patient-provider interaction (micro); and, ensuring the methods were feasible for sites to implement, respectively.

To ensure breadth and relevance, rehabilitation clinics from the single provincial health-system and private-provider sites were included. Geographical diversity was ensured by including sites from from rural areas (population < 10,000), regional-urban areas (population between 10,000 and 100,000), and metropolitan-urban areas (where population > 100,000). This study was approved by the Conjoint Health Research Ethics Board (University of Calgary).

Participants included current patients and providers visiting and working, respectively, at study sites. Provider inclusion criterion was employment at the site. There was no limit on type of healthcare provider discipline. Patient inclusion criteria included ≥18 years of age; their rehabilitation provider was participating; able to consent without proxy; and can understand and speak English. These criteria were driven by ethical and feasibility concerns. There were no exclusion criteria.

Convenience sampling, informed by site leadership, directed provider recruitment. Tactics included researcher’s email introductions followed by study presentations (by webinar, in-person, or one-on-one) overviewing study aims, methods and implications. Informed consent was obtained. Recruitment continued to saturation for providers per geographical area as feasible.

Convenience sampling directed patient recruitment. Trained, onsite receptionists or therapy assistants identified and recruited eligible patients, who then expressed interest in interview participation directly to researchers. Only patients of included providers were included to promote triangulation on the same patient-provider encounter from diverse perspectives and to minimize site burden. Recruitment continued to saturation for patients per geographical area as feasible.

We used unstructured, guided interviews to clarify communication experiences during appointments (supplemental file) [36–38]. We ask participants to describe appointments that went well and that did not go well, communication wise; providers were also asked to describe training they found to be influential to their work or any organizational or site traits that influenced them. The lead researcher (KPM), a researcher with legal and bioethics training alongside post-doctoral experience in qualitative research conducted all 1:1 interviews by phone or in-person, based on feasibility and participant preference. She had no previous relationship with participants. Interviews were audio-recorded and transcribed.

Data collection and analysis was simultaneous. Analysis began by uploading cleaned transcripts into NVivo. The lead researcher coded transcripts for words and phrases related to barriers to, and facilitators of, SDM. Similar ideas were grouped together to form themes, with tentative relationships among the themes identified. Using participant type and geographical area, the lead researcher separately analyzed six groups of transcripts. The three patient groups from each geographical area were incorporated into a larger patient-specific model. The same was done for provider insights. Patient and provider models together built the full framework.

We promoted the research rigour through several tactics [36]. Credibility was established through peer debriefing, member checking, and negative case analysis. We shared interim findings with organizational leadership, patient advisors and knowledge users to ensure resonance. During data collection and analysis, we consciously sought data supporting alternative explanations so that initially identified researcher assumptions did not direct results (i.e. were there other connections between SDM and outcomes) [36]. This study was preceded by a feasibility study; we had more than 1 year of connection with sites and leadership. This fostered engagement, trust and rapport. Transferability was addressed through use of thick description from interview transcripts and field notes to ensure contextualization.

Confirmability was achieved through use of audit trail, triangulation and reflexivity. The audit trail included raw data, data-analysis documents, and final deliverables. This audit trail yielded a clear research path examinable by the research team. Collecting data from a diverse sample of patients and providers from three geographical areas permitted triangulation, particularly our iterative levels of saturation. Reflexivity was promoted in team meetings with researchers briefly summarizing their personal learning as the study progressed. To ensure transparency, we exposed pre-existing, researcher perspectives in the proposal and field notes to promote team discussion [36, 39]. Finally, dependability was established through the availability of the audit trail to team members including a knowledge-user for assessment, to root out errors [36, 39].

## Results

We conducted 49 one-on-one interviews: 23 with patients, 26 with providers (duration 15 to 60 min each). Participants were recruited from 14 community-rehabilitation sites, three of which were private providers and 11 were from the single provincial health system. Geographically, three sites represented rural areas (*n* = 8 patients, *n* = 5 providers); five represented regional-urban sites (*n* = 10 patients; *n* = 8 providers); and six represented metropolitan-urban sites (*n* = 8 patients; *n* = 10 providers). The proportion of female participants was similar for patients (73.1%) and providers (73.9%). All but one patient participant visited rehabilitation for chronic conditions. Most of the provider-participants practiced physical therapy (47.8%) or occupational therapy (30.4%). The findings from the six sub-groups were brought together to develop an overarching framework to explore SDM experiences, as well as patient- and provider- perceived barriers and facilitators.

*The Spectrum of SDM Experiences in Community Rehabilitation.*

**Fig. 1:**
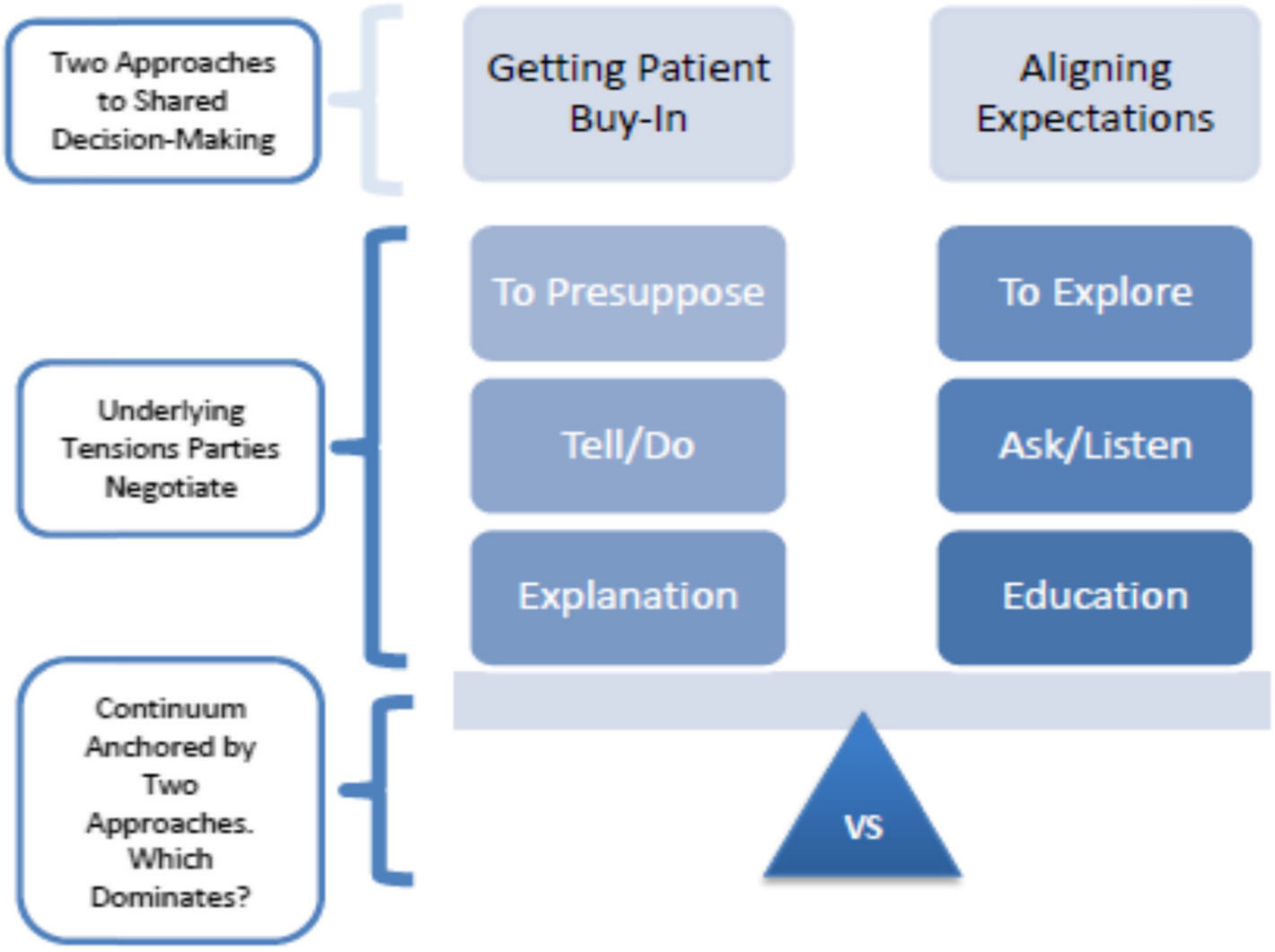
The Spectrum of SDM Experiences in Usual Care Community Rehabilitation

At these provincially-dispersed community-rehabilitation sites, SDM is present, but inconsistent in its appearance. SDM experiences appear to fall along a continuum anchored by two approaches: Getting Patient Buy-In and Aligning Expectations (Fig. 1). The former lacks flexibility, is provider-driven, and is prescriptive. The latter is collaborative, inquisitive and empowering. Aligning-Expectations contains the features of high-quality SDM.

In Getting-Patient-Buy-In, providers generally dominate and direct conversations, goals, and planning. Patients are less involved. The aim of interactions is patient adherence to provider-driven goals and plans.

*“I probably end up going into paternalistic mode because at the end of the day you’re like ‘well okay that’s the direction we’ll take it then if you’re not [sic] making any decisions.’”* [Rural Provider 1, Female]

“*I guess at this point I think I know what’s best for people but I always try to ask if we’re missing something. … I tell them you’ve got to let me know if this is not enough or too much or whatever it may be*.” [Regional-Urban Provider 4, Male]

*“I’m kind of a follower. I go along with what the treatment is. Yeah everywhere, I kind of trust the health providers.”* [Metropolitan-Urban Patient 1, Male]

In interactions that reflect Aligning-Expectations, communication is active, two-way, and mutually respectful. Patients and providers detail their rehabilitation wants and needs candidly towards clear goals.

***“****Well pretty much my goals are based [on] what their goals are. … I kind of tell them this is what we’re going to do to help achieve that goal and kind of what we’re doing [and] kind of my goal.”* [Regional-Urban Provider 2, Female]

*“Shared decision-making. This is what they’ve done. They said ‘Okay here’s the test. Let’s just try this, let’s just try this.’ Because for me, they had to prove to me that I wasn’t ready. And so because they proved to me that I wasn’t ready, I was motivated to become more ready.”* [Rural Patient 2, Female]

Three underlying dynamic tensions constitute each anchoring approach: (1) to presuppose versus to explore; (2) the language of tell and do versus the language of ask and listen; and (3) explanation versus education. Table 1 elaborates the facets of each tension, while Table 2 provides quotes supporting the tension. The interplay of these (not mutually-exclusive) tensions informed where an interaction fell on the SDM spectrum. An individual provider or patient was not wholly limited to one ‘spot’ on the spectrum (e.g. a single provider described an experience of telling the patient what to do, and another experience of approaching patients humbly).

**Table 1:** Tensions Underlying SDM Experiences in Community Rehabilitation

Tension	Meaning in Framework
To Presuppose **VS** To Explore	Preconceived assumptions stymie overtures and opportunities to learn from each other. Patients and providers seem to treat each other as obstacles to overcome not partners. Relationships lack traits of optimism, confidence, safety or motivation. **VS** There is awareness and divestment of preconceived notions. Collaboration is fostered by humility, inclusivity and feelings of safety. Awareness, availability, candour and respect typify interactions.
Tell/Do **VS** Ask/Listen	The provider mostly tells the patient what to do. Patients get few choices. Patients cannot, or will not, share their perspectives. Misunderstandings become frequent. **VS** Questions are posed. Responses are listened to and acted upon accordingly. Few limits (both internal and external) impede patient expression (e.g. ability to vent).
Explanation **VS** Education	Providers unilaterally share information to secure permission and adherence. Information is not personalized and at times confusing. Patients can be intimidated. **VS** Providers use knowledge to empower patients and support informed decision-making. Language is clear, consistent, realistic and patient-specific.

**Table 2:** Transcript Quotes Demonstrating the Underlying Tensions to the Extremes of SDM Experience in Community Rehabilitation

Underlying Tensions	Where an interaction falls along the above spectrum relates to an interplay of these (not mutually-exclusive) tensions for patients and providers (the parties).
To Presuppose **VS** To Explore	“Times [when] it doesn’t go well would be when maybe patient expectations and maybe my abilities don’t match particularly well at certain times.” [Rural Provider 4, Male] “I’m not working. And I’m paying all this money and I don’t feel like I’m getting better and I want to. … But how come nobody’s helping me to read again, or to work on a computer so I can do my job? So sometimes I think, she’s in a rush. And she’s hard to see and I guess she intimidates me a little bit.” [Metropolitan-Urban Patient 2, Female] **VS** “I find the assessment being a little more focused and a lot faster if I spend a lot more time on the introduction and the question and ask them what are they here for and explain what we can do.” [Regional-Urban Provider 2, Female] “What I was doing at work. … That was all taken into consideration. ‘What are you doing?’ … So, it’s working around that and creating a plan that is beneficial so that anything I do at work is not going to affect my rehab. And my rehab isn’t going to overload what I do at work, or vice versa. [Metropolitan-Urban Patient 4, Male]
Tell/Do **VS** Ask/Listen	“At this point I think I know what’s best for people, but I always try to ask if we’re missing something …. I tell them you’ve got to let me know if this is not enough or too much or whatever it may be.” [Regional-Urban Provider 4, Male] “There was some appointments that I felt like they were just doing what they had previously. … And I kept saying nothing’s really changing…. But that person never thought well maybe we should try something different. And I found that frustrating.” [Rural Patient 7, Female] **VS** “As a clinician I’m looking at everything from the clinical aspect right, but as the patient they’re looking more from the functional aspect. And I see the client only once a day probably for an hour, but they’re the ones who are living the day to day life. … So that’s where the shared decision is, we’re going to work on this, …this is going to help you in achieving this functional goal you have set for yourself, are you okay with that?” [Regional-Urban Provider 6, Female] “We’re always maybe a little disappointed with some of the other professions and some of the health staff at the lack of communication and the lack of putting out all the options to everybody. Even when they do have communication difficulties, they’re still able to make that decision right?” [Regional-Urban Provider 3, Female]
Explanation **VS** Education	“Sometimes I thought that they were understanding what I was saying whereas sometimes they’re not. They didn’t understand everything fully and you don’t know that sometimes until they come back.” [Rural Provider 3, Male] “So the only thing that I can say is that a more formalized plan would be … written down, [and it] would give you the ability to go back and say okay when you came to see me in January this year we were working on your back and neck and now we’ve moved to your shoulder and your pecs, so are these connected or are they not connected?” [Metropolitan-Urban Patient, Female] **VS** “So my best one’s are more where we kind of work together and have a conversation rather than just ‘okay I’m going to stand up and just lecture you on what you’re going to do.’ It’s all back and forth right even throughout the day. There’s lots of questions I’m asking, it’s not just on how does it feel…”[Male, Provider 4, Regional-Urban] “They’re all very good. They explain the bone or the muscle or whatever it is and what it does and what you should do and what you shouldn’t do, to try to let it heal.” [Regional-Urban Patient 1, Female]

The tension between presupposition and exploration turned on how open, collaborative and confident the patient and provider were with each other.

**To Presuppose**

*“Another really good one is almost like fortune-telling sometimes because they’ll tell you symptoms and then you can always already jump into other symptoms … they did not mention. … So they know what I’m talking about and they understand me and they trust my expertise.”* [Metropolitan-Urban Provider 3, Female]

**To Explore**

*“When I first started out, there would be a lot of me sort of being in my own head a lot and making sure I’m prepared, making sure I’m saying the right things, making sure you’ve done your research ahead of time. … But what I [sic] think has changed, and gradual more than anything, but it’s that just kind of being more in the moment and trying not to come up with what I’m going to do or say ahead of time and having [a] more organic sort of growth together.*” [Rural Provider 4, Male]

The tension around language turned upon whether the language used was uni-directional (where information flows in one direction) or bi-directional (where both patient and provider communicate effectively by sharing with, and hearing, each other).

**Tell/Do**

*“”[T]hey didn’t understand my reality. So I have nurses that come and dress me everyday. … If you take that shoe off of me, that’s fine. But then I need someone to put it back on me. So I mean it’s not their fault they would not have an idea of what my actual life is like.”* [Regional-Urban Patient 7, Female]

**Ask/Listen**

*“I need to understand what their concerns are and the goals and how we both will get there. And from my part, I need to listen and break it down and from their part is they need to follow-up with the exercises and or treatment implementation that I set so then we can talk about it together if that works for them. And sometimes it doesn’t.”* [Regional-Urban Provider 5, Female]

The manner and purpose of information sharing could empower the patient or provider alone (explanation), or both patient and provider (education). Explanatory information-sharing featured permission-securing purposes; incomplete understanding; and, lacked clarification of understanding. Educational information-sharing enabled patients and providers to clarify each other’s perspectives, expectations and preferences.

**Explanation**

*“If you’re going in for surgery, you don’t understand the surgical risks and what’s going to happen. You could be told but you still don’t really understand. So you want the surgeon who is the expert to tell you this is what you need, this is what we’re going to do. And that’s how I approach it here, I give them options but I tell them this is the best.”* [Male, Provider 2, Metropolitan-Urban].

*“Sometimes I thought that they were understanding what I was saying whereas sometimes they’re not. They didn’t understand everything fully and you don’t know that sometimes until they come back. And you have to re-explain the diagnosis. Or if it’s an exercise you want them to do, you have to show them again they didn’t quite understand it the first time so it would maybe communication where I hadn’t been as clear as I thought.”* [Rural Provider 3, Male]

**Education**

*“Information. So in my opinion, people tend to not do enough or they overdo it. And so they give you guidelines to follow and that really helped stay within the parameters of being healthy.”* [Regional-Urban Patient 3, Female]

“*There’s been a couple time where not too much was happening for a while. So, I said to the doctor, “I feel like things aren’t really moving ahead like I thought they were”. Then he’s explain to me why right now were trying to open things up, because right now you’re all hunched over so we need to open that up. And then when we progress to the next level, the strengthening will start improving things. And I guess I was okay with it. I didn’t need to see improvement every single time.”* [Metropolitan-Urban Patient 6, Female]

### Perceived barriers and facilitators to SDM

**Fig. 2:**
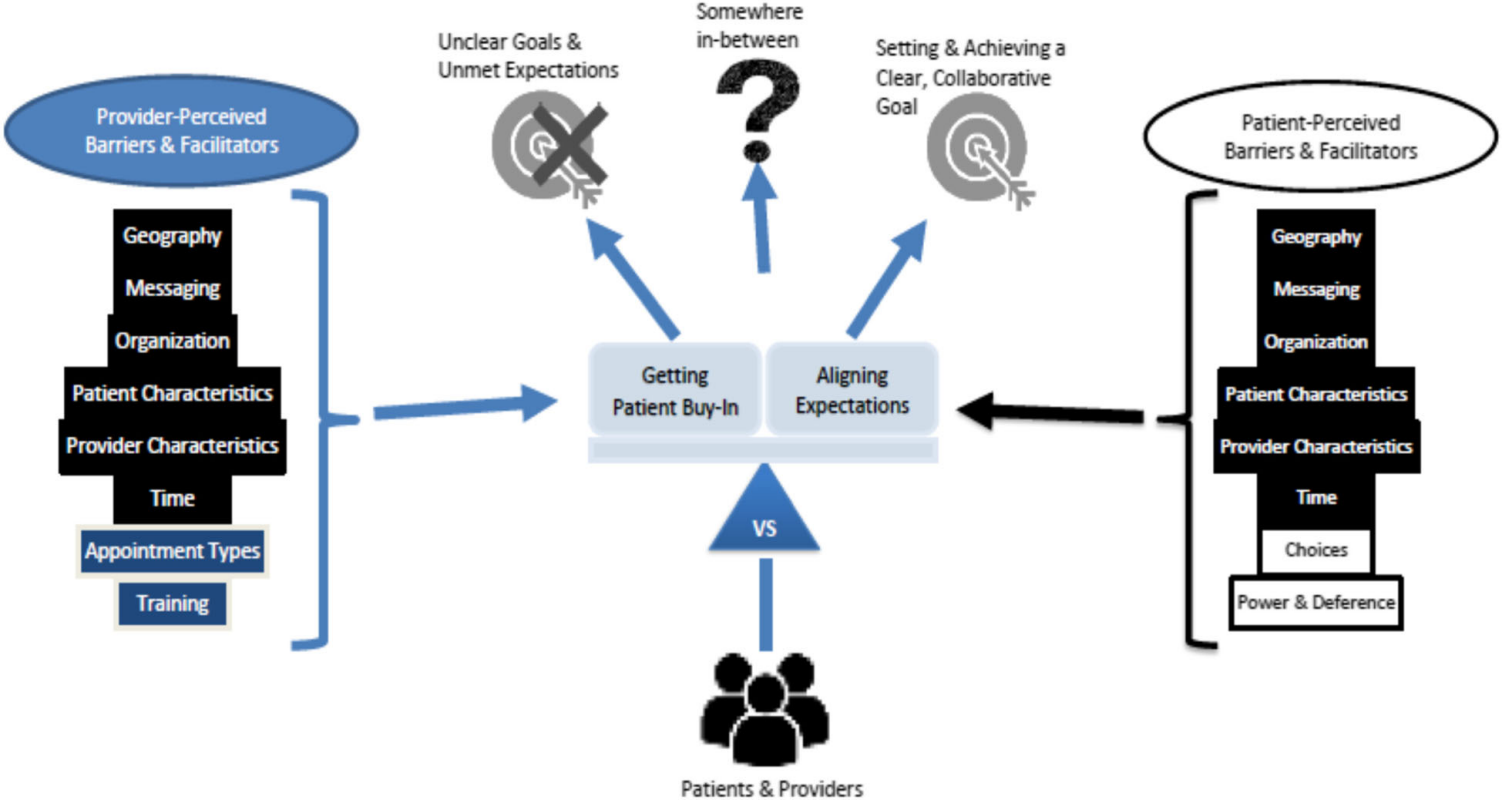
Barriers and Facilitators to the Experience of SDM in Community Rehabilitation

Patients and providers revealed factors that manifest as barriers or facilitators to the SDM experience in community rehabilitation (Fig. 2). These factors influenced how the underlying dynamic tensions manifest, and correspondingly where on the SDM spectrum a patient-provider interaction would fall. Tables 3 and 4 describe the SDM barriers and facilitators perceived by providers and patients, respectively. These tables contain a quote to exemplify the influential factor (Supplemental Tables 1 and 2 contain detailed quotes).

**Table 3:** Provider-Perceived Barriers and Facilitators in SDM in Community Rehabilitation as well as Exemplar Quotes

Provider-Perceived Barriers & Facilitators towards *Aligning Expectations* in SDM	
Geography	Place and distance accentuated other factors. Rural providers felt more control, independence and sense of community. Catchment areas affected long-term relations and goal realization. *“Then the other thing I would say that is different is that often times you have a relationship already with patients in rural setting. … It might be good. It might be bad. But for the most part it’s a lot of work is already kind of done before the person even walks in the door.”* [Rural Provider 5, Female]
Messaging	Providers felt SDM went better when using visuals; clear language; specific and simplified strategies tied to the patient’s circumstances and preferences. *“[H]ow strong and powerful words can be with certain again different genders or different cultures…. Just knowing that maybe somebody doesn’t have strong anatomy knowledge or medical knowledge, if you say certain words it can be very scary to them and that can exacerbate their pain.”* [Metropolitan-Urban Provider 1, Female]
Organization	SDM was promoted by available patient and organizational financial resources; more privacy during appointments; facilitative intake and eligibility processes; standardized resources; and, the strong, collaborative and available multidisciplinary relations and environments. *“It doesn’t happen as much in this clinic, but there’s also obviously financial limitations. Physio, chiro all of that stuff, it’s not cheap. People have benefits but usually doesn’t cover a full treatment course.”* [Metropolitan-Urban Provider 2, Male]
Patient Characteristics	Providers attached the SDM experience to patients’ modifiable and non-modifiable attributes, including age, lifestyle, capacity, emotionality, memory, realism, and perceived desire for control. Exemplar barriers manifest as older age, less-active lifestyle, and diminished capacity. *“I think different cultures can kind of play into that so if it’s maybe someone from a cultural background or they just didn’t really have an experience with exercise. I think with different sets of cultures, you see that they’re very much like you’re the healthcare practitioner you’re supposed to fix me, I don’t need to do the homework.”* [Metropolitan-Urban Provider 1, Female]
Provider Characteristics	Previous personal and professional experience promoted collaborative SDM and patient communication. Providers’ training, previous employment, disposition, confidence, and approach to culture were particularly influential. These factors populated underlying tensions. *“I think the turning point was when my dad had a stroke…. The physical deficits doesn’t affect me as much as the personality and the cognitive deficits I saw happening in him…. It’s not just about physical deficit, it’s the overall person, where they’re coming from, what is important to them. …* [Female, Provider 6, Regional-Urban]
Time	Perceptions of more time together facilitated SDM by promoting provider experience, rapport-building, and perceived patient acceptance. Externally, or self-imposed, time limits impeded communication and SDM. *“Because we get in habits a lot of the time when we’re a little bit short on time or we consider ourselves short on time. … The patients thinking about an answer, [and] sometimes we tend to jump in and try to help them answer it instead of just letting them have the time to do it.”* [Regional-Urban Provider 4, Male]
Appointment Types	Appointments vary by reason, group-vs-individual, and patient population. Collaboration felt limited in hand and equipment-approval appointments with physician-imposed plans and standardized processes. SDM was impacted by how providers navigated group camaraderie. *“I would say the things that often don’t go well is when a person has a sense of that they’re coming in for some sort of specific thing and that’s not what we’re able to provide. Let’s say a referral from a physician, and they have some expectation of what we’re able to do that’s not actually within our scope of practice.”* [Regional-Urban Provider 1, Female]
Training	Providers who participated in communication training (e.g. HealthChange®) spoke in the language of ask and listen, empowered patients via education and practiced respect to promote better SDM. *“HealthChange … I find the assessment a little more focused and a lot faster if I spend a lot more time on the introduction and the question and ask them what are they here for and explain what we can do.”* [Regional-Urban Provider 2, Female]

**Table 4:** Patient-Perceived Barriers and Facilitators in SDM in Community Rehabilitation

Patient-Perceived Barriers & Facilitators towards *Aligning Expectations* in SDM	
Geography	Patients often felt very comfortable with rural providers with longer relationships, sense of community and greater privacy. This facilitated SDM. Physical distance and lack of choice were geography-related barriers. *“That’s one real advantage of [Town 3] is that he is it. And there’s next to nothing else going on. There’s no other distractions. He’s focusing entirely on you. … He takes his time.”* [Rural Patient 4, Male]
Messaging	Patients prefer consistency in provider approaches and language at repeated appointments. Patients appreciated evidence of multidisciplinary collaboration and where follow-up specific to, and built upon, similar previous discussions. *“With the massage therapist, I feel confident, it’s an in-depth, specific conversation. It’s not ‘how are you today?’ He says to me every time, I read your notes of what you did with [the physiotherapist] last, have you seen the concussion specialist? What’s happening with that? … And then he says okay what’s the worst thing that’s a challenge to you this week, or today, and then he says, what would you like me to work on?”* [Metropolitan-Urban Patient 3, Female]
Organization	Organizational facilitators included feelings of privacy, family interactions, and availability of multiple, collaborative professionals. Barriers related to finances, waitlists, professional relationships, and service convenience. *Especially if I’m just put on a machine and it’s like here you go just pay $85. It’s like would it be cheaper if I just buy the darn machine and do it at home?”* [Metropolitan-Urban Patient 3, Female]
Patient Characteristics	Patients’ influential traits included level of assertiveness, lifestyle, positive perception, perceived responsibility, perseverance, self-deprecation, memory, emotions, and vulnerability. Facilitators included active lifestyles, positive attitudes, and taking personal responsibility. *“I am pretty coy about [my goals] you know. I think he recognizes [*sic*] that different people have different goals and motivation. Like I’m sure there’s lots of couch potatoes out there. In fact another fella that I know went to [the physiotherapist], he’s kind of accepted where he is and I’m not that kind of person.”* [Rural Patient 4, Male]
Provider Characteristics	Patients felt most comfortable with consistently-available and previously-known providers. Patients positively contrasted rehabilitation providers to other professions (physicians, dentists). *“[The previous physiotherapist] just kept insisting that we have to go through with it. And I’m like no … and he just never let up on it and finally I just wouldn’t go back anymore.”* [Rural Patient 3, Female]
Time	Time impeded SDM when patients perceived appointments as abrupt; issues as novel; experiences as unremarkable and unsustainable; and physical progress as too slow. Time-related facilitators afforded dynamism and apt appointment frequency. *“She talks very fast, she moves very fast, and it’s like, I feel like ‘oh we’ve got to get you out of here because there’s 500 other people waiting and I don’t have time for that.’ … And I don’t want to upset anybody. But [*sic*] this whole thing has made me a little insecure to voice my opinion.”* [Metropolitan-Urban Patient 3, Female]
Choices	The availability of choice was important, but not absolute. Convenience, accessibility and geography influenced this availability. Rarely, patients sought second opinions. *“We’re equal distance between [Town 1], [Town 2] and [Town 3] and so I could go any of those places. … So [Town 3] is convenient, but if it wasn’t the quality then I probably would be going elsewhere. So it’s convenient but I also have good rapport, trust and think I’m getting high quality care when I go to [Town 3].”* [Rural Patient 4, Male]
Power & Deference	Patients expressed great deference to providers, which led to difficulties in expressing goals or complaints. Patients often went without care or without fully expressing their preferences. *“Shared decision-making [is that] they’ll help me out. I don’t know, you’re talking to an old old lady.”* [Rural Patient 1, Female]

Patients and providers had six factors in common: geography, messaging, organization, patient characteristics, provider characteristics, and time. Providers recognized two further factors: appointment types and training. Patients recognized two unique factors: choices as well as power and deference.

While details are in Tables 3 and 4, we elaborate a few factors to clarify their dual manifestations. For example, geography could facilitate SDM because smaller communities were attributed with fewer competitors for provider time and attention during appointments and with longer, trusting relationships. Geography could sometimes impede SDM because smaller communities were associated with fewer choices for some patients and more physical distance to travel for rehabilitation supports, which was challenging when people faced physical health issues. However, where there was confidence and trust in SDM, patients often did not seek alternatives.

“*Because of where we are and the people we serve, both because of distance or because of people are back in the workforce and can’t come once a week for six weeks, so then we adapt that program a lot. We have that discussion about ‘does this work for you?*’ [Regional-Urban Provider 7, Female]

*“That’s one real advantage of [Town 3] is that he is it. And there’s next to nothing else going on. There’s no other distractions. He’s focusing entirely on you. … He takes his time.”* [Rural Patient 4, Male]

Another example includes time. Time facilitated SDM when providers gathered experience and confidence in their professional skills and spent more time with patients to build trust. Time impeded SDM when patients were unsatisfied with the ratio of physical progress to time receiving rehabilitation services or when patients or providers perceived less time available during appointments.

*“Pain relief motivates me and being able to do the things [that] I like to do without having any issues and driven by results. So when I see results from their actions as well as my actions, then of course that builds trust and motivation to continue working with the practitioner*.” [Metropolitan-Urban Patient 5, Female]

A third example includes messaging. Patients and providers appreciated clarity, specificity and remembrance in their communication. They felt that fostered better quality SDM. Use of visuals, clear language, and simple strategies that were tied to the patient’s previously-described circumstances, needs and wishes were of great value.

*“Depending on what part they’re not understanding, I’ll use different things. I use visual tools. I use models. I use pictures. I use analogies. I use descriptions. I’ve got an arsenal of them from over the years from how I describe things. If they’re a mechanic, I’ll compare it to a car. … If they’re a baker, I’ll compare it to not having the right ingredients.”* [Rural Provider 5, Female]

## Discussion

The perceptions of SDM of patients and providers in this study reveal that explanations of SDM could be described as falling along a continuum, where high-quality SDM in community rehabilitation involves active, two-way, mutually-respectful communication. Patients and providers perceived many similar barriers and facilitators to SDM in community rehabilitation. Our findings correspond to, and expand beyond, the scant literature from the rehabilitation context [24–26, 40, 41]. Previous literature reviews suggest very negative and limited SDM experiences in rehabilitation. In contrast, we demonstrate positive examples, and the factors promoting them across diverse settings.

There is overlap between the perceptions of community-rehabilitation patient of SDM barriers and those of patients from physician-focused encounters. A systematic review (*n* = 44 studies from mostly physician-patient encounters) demonstrated patient-reported SDM barriers fell into two groups: health-system organization and decision-making interaction [21]. Organizational barriers included time, continuity, workflow, and setting characteristics, while interactional barriers included pre-disposing factors, decision characteristics, interactional factors, power imbalance, presumptions regarding patient role, patients undervaluing themselves, communication style, trust, and preparation [21]. Our findings confirm that the nature of communicators, the message, and their setting are influential and important to the SDM encounter experience.

Many factors that could enable or impede SDM were perceived in common between patients and providers in this study. Their divergences are telling, but are rooted in power dynamics. For patients, power imbalances, and correspondingly deference, in favour of professionals, influenced perceived SDM quality and patient engagement. For providers, power-related dynamics also seemed at play. Here, power favoured organizations and other professionals (particularly physicians). Appointment types included power dynamics either because organizational infrastructure dictated the time available to an appointment or physicians had more control over the content, planning and communication of certain rehabilitation (e.g. post-hand surgery). Training involves education and information, which is often touted as a great equalizer in power imbalances. Providers appeared to recognize that as well.

Six actionable items emerged to promote more experiences of Aligning-Expectation in community rehabilitation. First, provider training, especially particular training on patient-centred care and behavior change (i.e. HealthChange® Methodology [42]), are associated with better-quality SDM encounters involving exploration (not presupposition), listening (not telling), and education (not explanation). Many providers mentioned that the tactics from the patient-centred care workshops influenced their patient interactions. This identifies an alternative, more impactful training process versus the train-the-trainer SDM-development activities completed previously in Europe [25, 26].

Second, further educational strategies should be developed and tailored to address provider-perceived factors related to patient and provider characteristics. For example, training can give providers strategies and tools to overcome barriers associated with patient attributes (e.g. patients lacking active lifestyle, lacking assertiveness, and being overly deferent to providers). Training can compensate for provider traits manifest as barriers (e.g. lack of experience, lack of confidence, or lack of humility). Patients may not be as fully forthcoming due to their deference to providers and the health system and that patients may have plans of their own. Training that helps providers make space for patients to share could promote patient candour around preferences and plans. Training alone is insufficient; sustainability post-training is critical. Training should support the establishment of communities of practice, where providers continue to share their SDM stories, successes and challenges to enhance teamwork around a common theme across the system and to promote and sustain SDM training and skills in a variety of settings and with variations in patient characteristics.

Third, patients and providers were clear on the nature of messaging required in information sharing attuned to Aligning Expectations: clear, simple, patient-specific language with temporal consistency and connections to previous conversations between patients, providers and the health system. Humble, open communication works well. Open communication is not open-ended; patients seem to want specificity and continuity in conversations. A broad open-ended question sometimes works against the provider, as it suggests a lack of remembrance.

Fourth, our research clearly demonstrates that both patients and providers have expectations for the rehabilitation journey. Honesty and candour about expectations help patients and providers have collaborative experiences that support patients in setting and meeting their personal rehabilitation goals. Laying out expectations as the relationship unfolds breeds transparency and eventually alignment, satisfaction and patient-centredness.

Fifth, lack of time manifests as a barrier, while perceived adequacy of time was a facilitator. These findings corroborate but expand previous findings related to SDM barriers in physician contexts [18, 20, 21]. The broader literature suggests that the critical aspect is the non-quantifiable facets of time (e.g. caring, not rushing, active listening and trust) rather than the solely quantitative measure of amount of time spent [43–46]. Many providers spoke of feeling in control over their time and being free to spend time after patient-centred care training [42], but did not explicitly describe appointment times being lengthened. Further exploration on variability in perceived adequacy of time may be required, as it may be that more meaningful dialogue occurs in the same (or less) time if providers are taught to enhance SDM. Policy tools should be assessed that could empower non-rural providers to feel independent and in-control of their schedules and appointment spacing as their rural counterparts do. Those feelings seem to transfer to patients and help both patient and provider have a non-rushed conversation. In this setting, developing a community of practice again has value to transcend rehabilitation settings while empowering providers.

Sixth, broader policy changes may be needed for other identified, influential factors. For example, using technology to promote choices for patients and providers in geographically-remote areas may overcome geographical barriers. But, this study indicates that when care is collaborative, patients do not seek out alternative choices and relish the unique features of the sole choice in their community. Also, there is a need to explore how humility, collaboration, education and the language of ask and listen can follow patients and providers through different appointment types beyond traditional 1:1 appointments. There is a trend towards more efficient use of group-based programming in community rehabilitation. Further strategies and subsidies may be needed to redress waitlists and financial barriers that limit patients’ abilities to attend rehabilitation on a timely basis.

### Limitations

We recognize limits of this study. First, there may be a selection bias and non-response bias. Perhaps only patients with an extreme experience (either good or bad) were interested in sharing their experience with the study, so they would differ markedly from the general patient-population experience. It was unlikely, given the difficulty in patient recruitment generally, to recruit non-responders to participate in a non-responder interview or survey. This study prioritized significant recruitment using convenience sampling to lessen the influence of these biases.

Second, there may have been a Hawthorne effect on providers wherein their knowledge of a study assessing their communication altered their communicative behaviours. Due to the importance of informed consent, this effect could not be avoided. All site providers were informed about the study and its focus on communication prior to recruitment. In Phase 1, it appeared that rehabilitation professionals were accustomed to being observed during practice given their own training, the multidisciplinary players, and trainee presence at many sites. One patient-participant in this study noted a marked difference in her provider’s communication style that felt connected to study participation. This seemed isolated and was not replicated by other patient-participants.

Third, while our sample was geographically diverse, involved different rehabilitation professional disciplines, and patients with diverse conditions requiring rehabilitation, we lacked representation from diverse ethnicities and cultures. This limitation was compounded due to lack of interpretation services. Future research in settings that care for diverse patients from different cultures and ethnicities would be valuable. Finally, we did not have access to full peer review of all interview transcripts. Patient co-investigators (JM, ST) independently transcribed a sub-set of transcripts, which led to a discussion on themes and relationships with the lead researcher (KPM) who coded all transcripts. Availability and costs prohibited more in-depth and independent second assessments on coding. Many other tactics were used to promote rigour including audit trail, thick description, and negative case analysis.

## Conclusions

We have found both positive and less-than-ideal experiences of SDM in community rehabilitation. Many distinct, but not mutually-exclusive, factors influence where a SDM experience falls along between two extremes. We proffer recommendations to advance high-quality SDM in community rehabilitation based on promoting facilitators and overcoming barriers.

## Supplementary information

**Additional file 1.**

**Additional file 2.**

## Data Availability

The datasets generated during and/or analyzed during the current study are not publicly available due to the inability to anonymize qualitative data and secure participant privacy, but may be available in highly-redacted form from the corresponding author on reasonable request.

## Abbreviation

SDM: Shared decision-making
