# Supplementary material for: Experiences of shared decision-making in community rehabilitation: a focused ethnography
Source: BMC Health Serv Res. 2020 Apr 19;20:329. doi: 10.1186/s12913-020-05223-4 (PMC7168887; doi:10.1186/s12913-020-05223-4)
Supplement: Supplementary file 1 — Additional file 1. [file 12913_2020_5223_MOESM1_ESM.docx]

# ASK-MI Community Rehabilitation PHASE 2: PATIENT INTERVIEW

INTRODUCTION

Thank you for joining our study. As noted in the consent form, your participation is entirely voluntary. Please feel free to skip some questions if you wish. The interview will take about an hour. If you would like to take a break for any reason during the interview, please let me know.

If not complete, PRE-INTERVIEW DATA COLLECTION SHEET: Please tell us about yourself? We can use this sheet as a starting point for that.

# INTERVIEW GUIDE

1. How did you decide to seek rehabilitation services, generally and at this clinic specifically?
2. Appointments with rehabilitation providers are a key part of the rehabilitation process. **Could you walk me through your first appointment at this community rehabilitation clinic?**
3. Thinking back over your appointments at this community rehabilitation site (or in community rehabilitation generally), **please describe a recent visit that went well from your perspective.**
4. **Please describe a visit (or set of visits) that did not go well from your perspective.**
5. Thinking over your recent appointments, can give an **example of how you and your provider came to a treatment plan or set of rehabilitation goals?**

- In particular, what happens after you describe the issue that brought you to see this provider?

5. **When you hear the term “shared decision-making”, what does that mean to you?**

- Studies show that the best health outcomes occur when individuals and their providers make treatment decisions together, and that people have varying ideas on the degree to which they like to be involved in decisions about their healthcare. We are interested in your position.

1. Thinking back to a therapeutic plan developed at a recent appointment at this clinic, please **describe what influenced your decision to follow, or not follow, the plan?** What, if anything, would have made it easier, or more desirable, to follow the treatment plan?

- Research shows that very few people actually follow medical advice all the time. We want to know where you stand, in most cases of your rehabilitation care.

1. Is there anything else that we haven’t discussed but that **you would like to share**?

**ASK-MI Community Rehabilitation: PROVIDER INTERVIEW**

INTRODUCTION

Thank you for joining our study. As noted in the consent form, your participation is entirely voluntary. Please feel free to skip some questions if you wish. The interview will take about an hour. If you would like to take a break for any reason during the interview, please let me know.

If incomplete, PRE-INTERVIEW DATA COLLECTION SHEET: Please tell us about yourself and your practice? We can use this sheet as a starting point for that.

# INTERVIEW GUIDE

Around (SHARED) DECISION-MAKING

1. When you have a new assessment, can you **walk me through the appointment process**?
   - What happens? Who is involved? Types of questions asked? What activities completed?
2. We are interested in your experience in making treatment decisions or plan with patients.
   - Please **describe a recent visit that went well from your perspective.**
   - Please **describe a recent visit (or set of visits) that did not go well from your perspective.**
3. **When you hear the term “shared decision-making”, what does that mean to you?**

- Studies show that the best health outcomes occur when individuals and their providers make treatment decisions together, and that people have varying ideas on the degree to which they like to be involved in decisions about their healthcare. We are interested in your position.

1. **How do you approach uncertainty with your patients**? How do you communicate uncertainty (whether in diagnosis, prognosis, treatment plans) to patients?
2. Is there anything else that we haven’t discussed related to decision-making with patients, that **you would like to share**?

Around ORGANIZATIONAL SETTING

1. Training and resources are offered in many different venues to influence how patients and providers interact around decision-making. **Could you please give an example of any training or resources that have been influential to your practice, especially around decision-making?**

- The influence could be positive or negative.
- If nothing has been influential, why do you think that is the case?
- Looking at your career so far, have there been a turning points around how you approach interactions with patients? Please describe those turning points.

1. How, if at all, **does the context in which you work influence your appointments and interactions** with patients?
   - Context can include the physical space, the people, the organization and infrastructure. **How do any of these influence how you interact with patients?**
   - **Have you worked in any other settings? If so, did context play a different role there?**
2. Is there anything else that we haven’t discussed that **you would like to share**?
