# Supplementary material for: Experiences of shared decision-making in community rehabilitation: a focused ethnography
Source: BMC Health Serv Res. 2020 Apr 19;20:329. doi: 10.1186/s12913-020-05223-4 (PMC7168887; doi:10.1186/s12913-020-05223-4)
Supplement: Supplementary file 2 — Additional file 2. [file 12913_2020_5223_MOESM2_ESM.zip › ThePatient SUPPL 1 qual SDM in comm rehab 092319R2.docx]

**SUPPLEMENTAL 1**

**Detailed Quotes from Providers on Factors Impeding or Facilitating SDM in Community Rehabilitation**

| **Barriers & Facilitators** | |
| --- | --- |
| Provider-Perceived Barriers & Facilitators towards *Aligning Expectations* in SDM | |
| Geography | “You kind of have to create boundaries for yourself right. And then like you can only do that yourself right. If I let it go, it would be that I’m working 24/7 right. But if I get questions in when I’m downtown or something like that, I will just get them to give me a call at work that type of thing. I try not to yeah engage in too much of that. But the odd time you might see somebody that you know is particularly vulnerable at the time you might spend a bit of time. But for the most part I kind of nip it in the butt yeah.” [Rural Provider 5, Male]  “Because of where we are and the people we serve, both because of distance or because of people are back in the workforce and can’t come once a week for six weeks, so then we adapt that program a lot. We have that discussion about ‘does this work for you?’ [Regional-Urban Provider 7, Female]  “I guess in rural I could be playing hockey. I was playing hockey with somebody on the weekend and I guess it was good because I was able to give them some advice that they should get some physio and get someone to look at it and I was able to help diagnose and get them some treatment and it ended up being a fairly positive influence. … And in other cases I guess, I haven’t really had it, but maybe different expectations, maybe wanting to get in sooner. I’ve had that maybe once or twice but nothing that’s really affected. I just had an impression maybe it was their expectation because personally knowing them or family members that maybe they would get almost preferential treatment… when they found it there’s a waiting list.” [Rural Provider 3, Male] |
| Messaging | “I’ll actually create a tracking sheet and so I’ll break it out into columns. So everyday, they’ll have a column and they have little ticky boxes. So we will write together, we will write out [sic] what are the exercises we want you to complete, how many repetitions do we want you to do it. So every time they sit down and they do their exercises, they will do a checkmark. So we’ll kind of use that as a tool to help with exercise compliance and adherence and sometimes patients choose just not to do it and in those scenarios I will be very up front with them and say well realistically if you’re not going to put in that effort, you’re probably not going to see a change in your outcomes because you do need to do the exercises to see and improvement.” [Metropolitan-Urban Provider 10, Female]  “I think we probably ask them everyday, usually when they first come in. We ask them ‘so how are you feeling?’, ‘what’s your pain level like?’ and then we ask them ‘how are their exercises going?’ And, then if, for example, say somebody wants to get back to running and if we kind of said like you can try running, we ask them how that went or whatever the goal is ‘have you tried this activity?’ or ‘how was [sic] your pain at work?’” [Metrpolitan-Urban Provider 1, Female]  “Depending on what part they’re not understanding, I’ll use different things. I use visual tools. I use models. I use pictures. I use analogies. I use descriptions. I’ve got an arsenal of them from over the years from how I describe things. If they’re a mechanic, I’ll compare it to a car. … If they’re a baker, I’ll compare it to not having the right ingredients. So using whatever their experience is to try and explain it further.” [Rural Provider 5, Female]  “I think the better you communicate and build rapport with patients, the better they will do. I used to think it was more based on physical skill and your treatment skills and abilities, but it’s not more than half that. It’s more relating to the patients and making them feel that they can get better and you care they get better.” [Metropolitan-Urban Provider 2, Male] |
| Organization | “Everything is context dependent. So it depends on their insight. It depends on their emotional regulations for that day. Depends on their language abilities whether they have aphasia, whether they have ESL, whether they have had much rehab before, what their understanding of what things are. But for most part, I would say for me probably 70% of the time or 80% of the time, I’m able to work with the person in establishing their goals in the first couple sessions. There’s always a 20, 30% you have more difficulty with everybody so I don’t think a system can fix that.” [Metropolitan-Urban Provider 8, Male]  “It doesn’t happen as much in this clinic, but there’s also obviously financial limitations. Physio, chiro all of that stuff, it’s not cheap. People have benefits but usually doesn’t cover a full treatment course. So that kind of definitely comes into play.” [Metropolitan-Urban Provider 2, Male]  “Maybe just overbooking. Like we got to make sure that we’re not too busy that we’re still able to attend to patients and goals and not do rushed treatments. So we focus on that here. So we’re still obviously busy. It’s a business, we got to be financially stable but not to the point that we’re not doing as good of a job as we can for patients.” [Metropolitan-Urban Provider 2, Male] |
| Patient Characteristics | “Right so there are certain patients that expect that one visit or two visits is going to completely take care of their issue, even though I try to communicate to them ‘that’s not a super quick fix.’ Sometimes especially if it the condition is chronic or long-standing. … I guess those are the patients that are challenging, where maybe after the first one or two visits they haven’t seen a lot of improvement but then they [sic] don’t want to continue coming.”[Metropolitan-Urban Provider 5, Female]  “I think different cultures can kind of play into that so if it’s maybe someone from a cultural background or they just didn’t really have um an experience with exercise or with physio in general. And sometimes I think with different sets of cultures, you see that they’re very much like you’re the healthcare practitioner you’re supposed to fix me, I don’t need to do the homework. … I find those can be challenging. And I get people who are not as body aware as other people, so when you do show them an exercise or you show them something they find it very challenging and hard to do and as much as you try to correct it, they’re just very body unaware it’s a challenge.” [Metropolitan-Urban Provider 1, Female]  “I feel it hasn’t gone well: it’s when you can kind of tell that the patient is very skeptical from the get go. …So often I feel like your early patient attitude will influence [sic] whether or not that interaction will go well and often whether they continue to pursue care or if they go elsewhere. … Sometimes it’s it feels like you’re fighting a losing a losing battle with them.” [Metropolitan-Urban Provider 5, Male]  “When they go well, the patients [are] usually pretty involved I think. And they’re motivated to get better and they’re [sic] want it. They show an interest in knowing what’s going on.” [Female, Provider 4, Metro-Urban]  “It’s very hard to prod some people I guess into the direction of what would you like to do, what do you want to work on, like they kind of look at you and go ‘I don’t know you’re the professional you tell me.’ I’ve even heard people say that right. So it’s all well and good to say that we want them to make the decisions, but they’re looking to us for and you try to guide them and still make it their decision. But yeah and then of course with the cognitively impaired it’s virtually impossible.” [Rural Provider 1, Female] |
| Provider Characteristics | “I think my training has just been gained mostly from my experiences. And then I think … maybe being an occupational therapist is there’s going to be a heavy emphasis on communication and kind of holistic thinking. And there’s a component of mental health that we learn a lot of training on.” [Rural Provider 5, Male]  “This is just my third year. So I’m fairly new. I definitely have had I guess in my earlier days, there’s definitely been some more challenging patients. Again whether that’s language barrier or cultural backgrounds or age or whatever it is, I think it’s harder to communicate. And I think with those challenging ones, you do kind of learn what did work for communicating or what didn’t. … I think now that I’ve had few years of experience again I think I know how to approach those people better.” [Metropolitan-Urban Provider 1, Female]  “I would think that this particular therapist would be quite good for someone who’s not as forthcoming because … he’s very respectful. … You know he’s not a powerful, forceful therapist. He’s very good at what he does, but he’s not a demeaning sort of person. He’s very good. So I think that for most people, going in they would find that they weren’t overwhelmed and they were treated with respect.” [Rural Provider 5, Female] |
| Time | “As a new grad, I was way more prescriptive and I was in an acute setting. I think now using like the COPM definitely has helped [sic] with the shared decision-making process and asking people what specifically in their home life and in their personal life and their everyday life has changed since their brain injury. … It makes it more functional. It’s not just like okay I need to do this word puzzle. Well if you didn’t do word puzzles before, maybe you don’t want to do that. Right?” [Metropolitan-Urban Provider 9, Female]  “Whether it’s stroke patients or [other] patients, … that’s what again within time limits. I know that some of my colleagues work in areas that there’s much more time restraint and there’s less time of that the person is getting discharged in a day and we have to get them walking on crutches [sic] or walking a lot, we have to be able to get them ambulatory. … That’s just the reality [that] they’re going to be out on the sidewalk, so they need to learn that so there’s not much room there for ‘what would you like to do today.’” [Regional-Urban Provider 7, Female]  “Probably when you have a really busy schedule and [it] doesn’t seem like there’s enough time to cause or there’s a complex problem where it’s really taking up your whole time slot [that] you’ve allotted for the patient. So probably the time constraints are the most challenging.” [Metropolitan-Urban Provider 5, Female] |
| Appointment Types | “We have two plastic surgeons here. And one of them [sic] when they send an order, we often will only see the client once. So, it does happen a bit more regularly because that doctor will say I just want this person to wear this splint for a few weeks and he won’t be following up. … We say if you’re having problems with your hand, you can phone and see, you can come in for treatment but it’s not the expectation that we’re discharging the splint and starting exercises and that sort of thing we typically are only seeing them the once. So out of our two plastic surgeons, 50% of the time essentially we’re only seeing the client maybe one or two times.” [Regional-Urban Provider 8, Female]  “I would say the things that often don’t go well is when a person has a sense of that they’re coming in for some sort of specific thing and that’s not what we’re able to provide. So sometimes let’s say receive a referral from a physician or something and they have some expectation of what we’re able to do, but that’s not actually something that’s within our scope of practice like ordering further tests [sic], the wrong kind of chest orthotics or something like that.” [Regional-Urban Provider 1, Female] |
| Training | “The two day course was good. It gave me a lot. I’ve taken a lot of motivational interviewing courses too so it was an extension of that, but it gave me some practical tools and a framework.” [Metropolitan-Urban Provider 8, Male]  “Definitely I would say so. We’ve been taking this … what’s it called, HealthChange methodology. Everyone had to take this course. … They really reinforce like shared decision-making, communication with patients and really just patient-centred care, so I think that’s pretty influential especially around people who maybe aren’t as familiar with that stuff. But definitely the program that I took in school was really focused on patient-centred care, family-centred care and really you’re doing what the patient wants not necessarily what you want.” [Regional-Urban Provider 5, Female]  “I also went to a conference. It was put on by the healthy living program here in medicine. …[the speaker] had done a talk on letting our clients drive the bus. And what I really enjoyed about this talk was she, the first half, the whole morning she turned it on us as healthcare professionals and she really dove into our feelings and how sometimes we naturally have these fix it feelings right, where we naturally want to fix it for people. And how we need to be in touch with those feelings and we need to learn to kind of let those feelings go so that our client can make their own decisions. … And again she went back to values right? So if we can be into our own values as a clinician that we can maybe empathize or learn to see our client’s values. … I also I had gone to another training with a psychologist in town. She had put it on for the workers. She was trained to give a presentation based on the Daring Greatly work by Brené Brown. And that again explores values and shame.” [Regional-Urban Provider 3, Female] |
