# Supplementary material for: Experiences of shared decision-making in community rehabilitation: a focused ethnography
Source: BMC Health Serv Res. 2020 Apr 19;20:329. doi: 10.1186/s12913-020-05223-4 (PMC7168887; doi:10.1186/s12913-020-05223-4)
Supplement: Supplementary file 2 — Additional file 2. [file 12913_2020_5223_MOESM2_ESM.zip › ThePatient SUPPL 2 qual SDM in comm rehab 092319R2.docx]

**SUPPLEMENTAL 2**

**Detailed Quotes from Patients on Factors Impeding or Facilitating SDM in Community Rehabilitation**

| Patient-Perceived Barriers & Facilitators towards *Aligning Expectations* in SDM | |
| --- | --- |
| Geography | “When you go to the physio, all the equipment and everything that they have there for you to work with, I’m sure that is the same in any hospital that you go into because they’re interested in seeing you get back to your life. … And I know that like we’ve never had to deal with physio in another centre but with my husband, we have had to be in different centres for his treatment. And we’ve run into wonderful people everywhere and the care is always consistent.” [Regional-Urban Patient 2, Female]  “For me because I live remote, an hour and half drive to come to the [City 1] center, it would have been nice if all of my appointments were on the same day. … I really enjoyed [one group session] but it was on another day so in the month of October, I had to find a way to travel into the city three days a week so that it was difficult. My husband would bring me to my son’s place in [Town 4] most of the time, and then my son would drive me in, and then my husband would come and pick me up the next day kind of thing. …. It’s really difficult for people who live remote to do this.” [Rural Patient 2, Female] |
| Messaging | “So now when I set a goal, it’s not I want to play golf by the end of the summer. … I’ve moved into a townhouse that has stairs. I want to be able to go up and down the stairs without hurting myself. I want to learn how to lift things so that I can lift something on my own and not be dependent on people. So my goals are changing to what can I do right now and in the near future. And if I start getting stronger and stronger then I can say, how would it look to set a goal to, can I try golfing, can I try going to a driving range do you think that’s a good idea?” [Metropolitan-Urban Patient 3, Female]  “The conversations are good because they encourage you. The exercises are good because they also encourage you to try more and do more.” [Regional-Urban Patient 9, Male]  “Because they did give me handouts on some of the exercises. And the other ones they had me do them once or twice, then I knew exactly what I was doing and was able to follow-up on them.” [Regional-Urban Patient, Female 2]  “They talk in simple terms. Anybody can understand. Not trying to confuse or cloud you with any kind of medical terms or anything. If there is anything like that, it’s explained what’s going on.” [Metropolitan-Urban Patient 4, Male]  “He’s there. He’s got his file open. He’s reviewed it before you come in and so I feel we’re on the same page and close. And he knows me, I know him. It’s a personal relationship in that sense and one-on-one no other distractions in the room and that’s one real advantage of [Town 3] is that he is it.” [Rural Patient 3, Male]  “What makes it good for me is if it ’s not rushed and they’re talking five million miles an hour and you can’t understand them, I hate that. So if they’re clear and taking their time and explaining the why behind things works best for me. … Why this would help or why that’s happening, that sort of thing.” [Metropolitan-Urban Patient 6, Female] |
| Organization | “The only thing is you see before you used to be able to go in, set up an appointment. Now you have at the [local] hospital anyways, I guess they call it like a walk-in and it’s first come first serve for your first appointment. And that I think it was 6:45 is when they start the walk-in and then you go and you take a number and then at 8 o’clock they start calling your number so of course everybody rushed there right a quarter to 8. But I like early appointments. The only thing is that I don’t care for that system. But once you’ve done your first walk-in then you make appointments after that. [Regional-Urban Patient 1, Female]  “My motivations are the same because I want to get better. I wouldn’t be paying all this money. It’s a lot of money. I have no coverage any more, I had very little coverage to start. So I’m paying a lot of money to get better and I wouldn’t do that if I didn’t feel it was super important. Sometimes I have to say I feel like the appointments are a waste of money. Especially if I’m just put on a machine and it’s like here you go just pay $85. It’s like would it be cheaper if I just buy the darn machine and do it at home? … Like when they’re hands on and they’re like okay let’s do this for the next week and we’ll check on it. Sometimes that really makes a difference because I come home and do the exercises.” [Metropolitan-Urban Patient 3, Female] |
| Patient Characteristics | “Maybe each week just spending a little more time catching up. …I’m the kind of person that if I have a question, I’m going to ask it. … They’re not asking us always to say how have you been doing, what’s this you know, or … do you got any questions. But they certainly are open to it.” [Regional-Urban Patient 5, Female]  “So I never overtaxed myself. I was aware of not overstressing my brain too much. And … the original brain specialist that I saw, he was quite impressed that I was able to self-monitor that way because he said a lot of people aren’t, they don’t self-monitor and they either hurt themselves or they get frustrated and they stop. … But, for me that wasn’t an issue, I knew what my limitations were on a day to day basis and [the physiotherapist] had some me that too… that some days you’ll be good and some days you won’t.” [Rural Patient 2, Female]  “I am pretty coy about it you know. I think he recognizes [sic] that different people have different goals and motivation. Like I’m sure there’s lots of couch potatoes out there. In fact another fella that I know went to [the physiotherapist], he’s kind of accepted where he is and I’m not that kind of person. I want to recover to the maximum that I know I can or that [the physiotherapist] feels I can. …I mean he’s the pro so he’s the one who should be able to say yes this is possible.” [Rural Patient 4, Male] |
| Provider Characteristics | “I went in for a physio appointment, and the physiotherapist was quite busy, there was quite a few people in there. But what I liked about it was he came over, he talked to me, he asked how I was doing. He checked up on the exercises he had given me previously. And then, he wanted the athletic therapist to work on my neck specifically before he did something. So I was really impressed that he gave over to somebody’s skill set that was better in that area that he was. I thought well that takes a lot … instead of him telling me he was going to do it and having him do it not as well, he had the right person do the right job. So I was impressed with that and walked away feeling good about my treatment that day. [Metropolitan-Urban Patient 3, Female]  “I’m still doing physio with the [therapist] so it was positive in the sense that well actually there is a bit of history here.” [Rural Patient 4, Male] |
| Time | “I find as I’m getting older and crankier, I’m getting better. How I used to always approach it is I’d go into the doctor and say this is my complaint and then they’d either do something about it or they’d put you off. And if they put you off, then I just wouldn’t go back for that complaint, I just wouldn’t bring it up again. I’d just live with it. And I think now I’m feeling like that’s not working for me anymore, you know.” [Metropolitan-Urban Patient 6, Female]  “Pain relief motivates me and being able to do the things [that] I like to do without having any issues and driven by results. So when I see results from their actions as well as my actions, then of course that builds trust and motivation to continue working with the practitioner.” [Metropolitan-Urban Patient 5, Female]  “No, this is now going on four months. And I think they’re just getting ready to discharge me because they feel like they tried everything and it’s not gotten any better. So I think it’s just kind of … like they discharge you when they feel appropriate, but I don’t think it’s like a limit on what like how many times you go.” [Rural Patient 7, Female]  “I think one of the funny things [is that] I’ve known that person for 20 years. I think one of the things is I think she does pay a lot more attention to from when I’ve been there years before. I think she listens when in massage when in the level of pain tolerance you can take getting massage in certain areas. Sometimes her massage is pretty intense. She was listening to me ‘I think that’s a little much, can we go a bit softer on the you know’ when she was trying to push some of the muscle fibres around cause it was pretty painful and then she backed off. [Rural Patient 6, Male]  “And the physio was the same. It’s probably one of the best ones I’ve been to. He’s explained things very clearly, gets in there, doesn’t waste a lot of my time. We’re not wasting. Like a lot of times in physio you go and they’re doing ice or heat, dragging it out because they’re running between five million patients at the same time. [Metropolitan-Urban Patient 6, Female] |
| Choices | “We’re equal distance between [Town 1], [Town 2] and [Town 3] and so I could go any of those places. And I have in fact for the sports medical or physiotherapy in at the university … I would just go across campus for an appointment. And then for a year or so after, I also went all the way into the city. So [Town 3] is convenient, but if it wasn’t the quality then I probably would be going elsewhere. So it’s convenient but I also have good rapport, trust and think I’m getting high quality care when I go to [Town 3] so those are all factors in the decision.” [Rural Patient 4, Male]  “And I’ve been so blessed in all of this. I couldn’t, if I had to hand pick the doctors and the support people that I’ve had, I couldn’t pick better people. And it’s all just luck of the draw that I got these people. … I could have got, there’s two physiotherapists in [Town 6], I could have got a different one. There’s I don’t know how many occupational therapists here at [City 1], I could have got a different one. I could have got different people at every step and I was so blessed to have the best of the best. It’s just absolutely, incredibly amazing.” [Rural Patient 2, Female] |
| Power & Deference | “I don’t know because they had it inside their heads. … They watch me walk and they watch me do the tasks I want to do and they kind of see from in their educated way what I am not doing that my body needs to do or what repairs that can be done. So I mean that’s a question that is way beyond my educational skill.” [Regional-Urban Patient 7, Female]  “Because these people are the professionals and I’m out of my element. And then to know that this woman is sought after for concussions, I don’t want to say to her ‘I don’t know what you’re doing for me. I’ve seen you five times now, or four times now, and I don’t see any improvement in my being able to work, or in my headaches. So what are you doing for me?’ Because I don’t want anyone to get defensive or upset because she’s the specialist.” [Metropolitan-Urban Patient 3, Female] |
